# Field attraction of *Eurydema ornata* (Hemiptera: Pentatomidae) to allyl isothiocyanate

**DOI:** 10.1038/s41598-023-37705-w

**Published:** 2023-07-08

**Authors:** Sándor Koczor, Miklós Tóth

**Affiliations:** Department of Chemical Ecology, Plant Protection Institute, Centre for Agricultural Research, ELKH, Budapest, Hungary

**Keywords:** Chemical biology, Ecology, Zoology

## Abstract

Several *Eurydema* species (Hemiptera: Pentatomidae) are considered as pests, however, reports on their chemical ecology are scarce. In the current study we focused on *Eurydema ornata* (Linnaeus) a pentatomid pest of several brassicaceous crops. Since the species is known to feed preferably on generative parts of plants, a series of floral and green leaf volatiles were tested by electroantennography and compounds eliciting remarkable responses were also tested in the field. Three compounds elicited the most outstanding responses from antennae of *E. ornata*: allyl isothiocyanate, phenylacetaldehyde and ± linalool. Field experiments were conducted in Hungary between 2017 and 2021 to test the potential attractive effects of the compounds. Three *Eurydema* species were caught in the experiments *E. ornata*, *E. oleracea* (Linnaeus) and *E. ventralis* Kolenati. In the experiments combinations containing allyl isothiocyanate attracted both males and females of *E. ornata*. The compound was also attractive on its own, in a positive, dose-dependent manner. When presented alone, neither phenylacetaldehyde nor ± linalool was attractive to the species, furthermore, addition of these compounds to allyl isothiocyanate did not affect attraction considerably. To our knowledge this is the first demonstration of field attration of an *Eurydema* species to a semiochemical and one of the few reports on trapping of a pentatomid species with a synthetic plant volatile in the field. Perspectives regarding research and potential practical applications are discussed in the paper.

## Introduction

Stink bugs (Pentatomidae) is a species rich family of true bugs (Heteroptera). Several pentatomids are considered as pests, and some may cause severe damages to crops^1^. Knowledge of the chemical ecology of pests is important, as it may provide tools for agricultural practice (e.g.^2^).

Recently, Weber et al.^3^ published a comprehensive review on the chemical ecology of Pentatomoidea. Studies on chemical ecology of Pentatomidae primarily focused on pheromone composition, however, for a lot of presumed pheromones information on their field activity is missing^3,4^. Furthermore, for several pentatomid pheromones it was found that bugs are attracted only to the vicinity of the traps, but they do not enter^4^. Beside pheromones plant volatiles may provide possibilities as attractants of insect pests (e.g.^5^). However, reports on attraction of pentatomids to plant volatiles are scarce^3^.

The genus *Eurydema* (Hemiptera: Pentatomidae) includes several, in general oligophagous pentatomids, which primarily feed on cruciferous plants, several species are considered as pests^6–11^.

Despite their importance, very few studies have been published concerning the chemical ecology of *Eurydema* spp. Aldrich et al.^12^ identified compounds from metathoracic scent glands of males of *E. ventralis* Kolenati and *E. oleracea* (Linnaeus), but behavioral responses were not recorded. Other studies focused on host plant choice of *E. ornata* (Linnaeus) and *E. pulchrum* Westwood^13,14^. Although the authors found differences in preferences for different host plants, no semiochemicals were identified in these studies. To our knowledge to date no reports on behavioral response of *Eurydema* spp. to semiochemicals have been published.

*E. ornata* is an important pest of cruciferous crops and caper^6,8,11,15^. The species was found to feed preferably on generative parts of plants^11,16^.

In our study we conducted electroantennographic screenings and field experiments on *E. ornata* in search for plant volatiles as potential attractants.

## Materials and methods

### Collected insects

Insects for electrophysiological studies were collected in the field with a sweep net at Halásztelek (Hungary). The collected individuals were taken to the laboratory and determined to species with a stereomicroscope, based on the works of Benedek^17^ and Derjanschi and Péricart^18^.

In the laboratory *E. ornata* adults collected were maintained in cylindric glass jars (diameter: 12.5 cm, height: 18 cm) on cauliflower sprouts on room temperature. Before the electrophysiological tests individuals were sexed and kept individually in glass vials.

### Electrophysiology

All chemicals for electrophysiological tests and field experiments were obtained from Sigma Aldrich Kft. (Budapest, Hungary). For each compound 1 mg/ml hexane solutions were prepared. Stimuli for electroantennographic (EAG) screenings were prepared as follows: 5 µL of the test solutions were loaded on 10 mm diameter Rotilabo filter discs (RKTech Kft., Budapest, Hungary) inside a Pasteur pipette.

In accordance with the observation that *E. ornata* prefers to feed on generative parts of plants^11,16^, the series of tested stimuli included several floral volatiles along with green leaf volatiles and allyl isothiocyanate, a characteristic compound of brassicaceous plants, which was found attractive to several pestiferous insects with cruciferous host plants (e.g.^19^). During the screening myrcene was used as a standard and was applied before and after the tested stimuli. Tested stimuli also included solvent control, in which only hexane was loaded on the filter disc and blank control, containing only an unloaded filter disc.

For electrophysiological tests, the antennae of live *E. ornata* adults were cut near the base and were mounted between glass capillaries containing Ringer solution. One of the electrodes was grounded while the other was connected to a high impedance DC amplifier (IDAC-2, Ockenfels Syntech GmbH, Buchenbach, Germany).

For presenting the stimuli to the antennae, a stainless steel tube (teflon coated inside) with a constant humidified airflow was set up. Stimuli were presented by a stimulus controller (CS-55, Ockenfels Syntech GmbH, Buchenbach, Germany), with a 0.5 s pulse duration. Measurements were made with the GC-EAD sofware (Ockenfels Syntech GmbH, Buchenbach, Germany). Stimuli were administered at ca. 20–30 s intervals. For evaluation of results, response amplitudes were normalized against the response means of the standard.

### Preparation of baits

Baits were prepared as follows: individual compounds, or blends of compounds were loaded on cotton wicks in polyethylene vial dispensers with lid (No. 730, Kartell Co., Italy). In Experiment 1 and 2, load of individual compounds was kept at 100 µL, both if loaded individually or in combination. In the dose–response experiment (Exp. 3) 1, 10 or 100 µL of allyl isothiocyanate was loaded in the dispensers. The lids of loaded dispensers were closed and for easier handling dispensers were attached to 8 × 1 cm plastic handles. Baits were wrapped singly in pieces of aluminium foil and stored at −18 °C until used. In the field baits were replaced after 4–5 weeks, as previous experience showed that the baits do not lose their activity during this period^20^.

### Field experiments

All field experiments were conducted at Halásztelek (Central Hungary) at an adandoned field with wild cruciferous plants. For field testing of synthetic compounds, CSALOMON^®^ VARb3 funnel traps were used (produced by the Plant Protection Institute, Centre for Agricultural Research, Budapest, Hungary). A small piece (1 × 1 cm) of household anti-moth strip (Chemotox^®^, Sara Lee; Temana Intl. Ltd, Slough, UK; active ingredient 15% dichlorvos) was placed in the containers to kill captured insects.

In the experiments treatments were set out in a randomized complete block design, with 5–8 m distance between traps. To avoid positional effects, trap positions were rotated regularly, as a rule on a fortnightly basis.

Based on electroantennographic results, allyl isothiocyanate, ± linalool and phenylacetaldehyde were chosen to be tested in field experiments. Treatments of field experiments are listed in Table 1.

**Table 1:** Treatments of field experiments.

Treatment	Exp. 1	Exp. 2	Exp. 3	Exp. 4
Allyl isothiocyanate	–	+	+^a^	+
± Linalool	–	+	–	–
Phenylacetaldehyde	–	+	–	–
± Linalool + phenylacetaldehyde	+	–	–	–
± Linalool + allyl isothiocyanate	+	–	–	–
Phenylacetaldehyde + allyl isothiocyanate	+	–	–	+
± Linalool + phenylacetaldehyde + allyl isothiocyanate	+	+	–	–
No bait	+	+	+	+

^a^In Exp. 3 dose–response to allyl isothiocyanate was studied.

In Experiment 1 binary and ternary combinations of ± linalool, phenylacetaldehyde and allyl isothiocyanate were tested along with unbaited, control traps. The experiment was run at Halásztelek, from 17th May to 11th September, 2017, with four blocks.

In Experiment 2 ± linalool, phenylacetaldehyde and allyl isothiocyanate were tested alone and in ternary combination along with unbaited, control traps. The experiment was run at Halásztelek, from 5th August to 11th October, 2018, with 5 blocks.

In Experiment 3, 1, 10 and 100 mg doses of allyl isothiocyanate and unbaited traps were tested. The experiment was run at Halásztelek, from 5th July to 25th September, 2019, with 5 blocks.

In Experiment 4, traps baited with allyl isothiocyanate, allyl isothiocyanate + phenylacetaldehyde and unbaited traps were tested. The experiment was run at Halásztelek, from 30th June to 5th October, 2021, with five blocks.

Trapped insects were collected on a weekly basis and were brought to the laboratory, where the individuals were sexed and determined to species based on the works of Benedek^17^ and Derjanschi and Péricart^18^ with a stereomicroscope.

### Statistics

Data were tested for normality by Shapiro–Wilk test. Since none of the experimental data were normally distributed, nonparametric tests were used. In field experiments catch data of individual traps were summed for trap rotation periods. Periods with low catches, accounting for less than 5% of total catches of the respective experiment were excluded from the analysis. Data from electroantennographic screenings and from field experiments were analyzed by Kruskal–Wallis test, and differences between treatments were evaluated by pairwise Wilcoxon test with Benjamini–Hochberg correction. For the dose–response test, Spearman’s rank correlation was calculated. Statistical procedures were conducted using the software R^21^.

## Results

### Electroantennographic screenings

**Figure 1:**
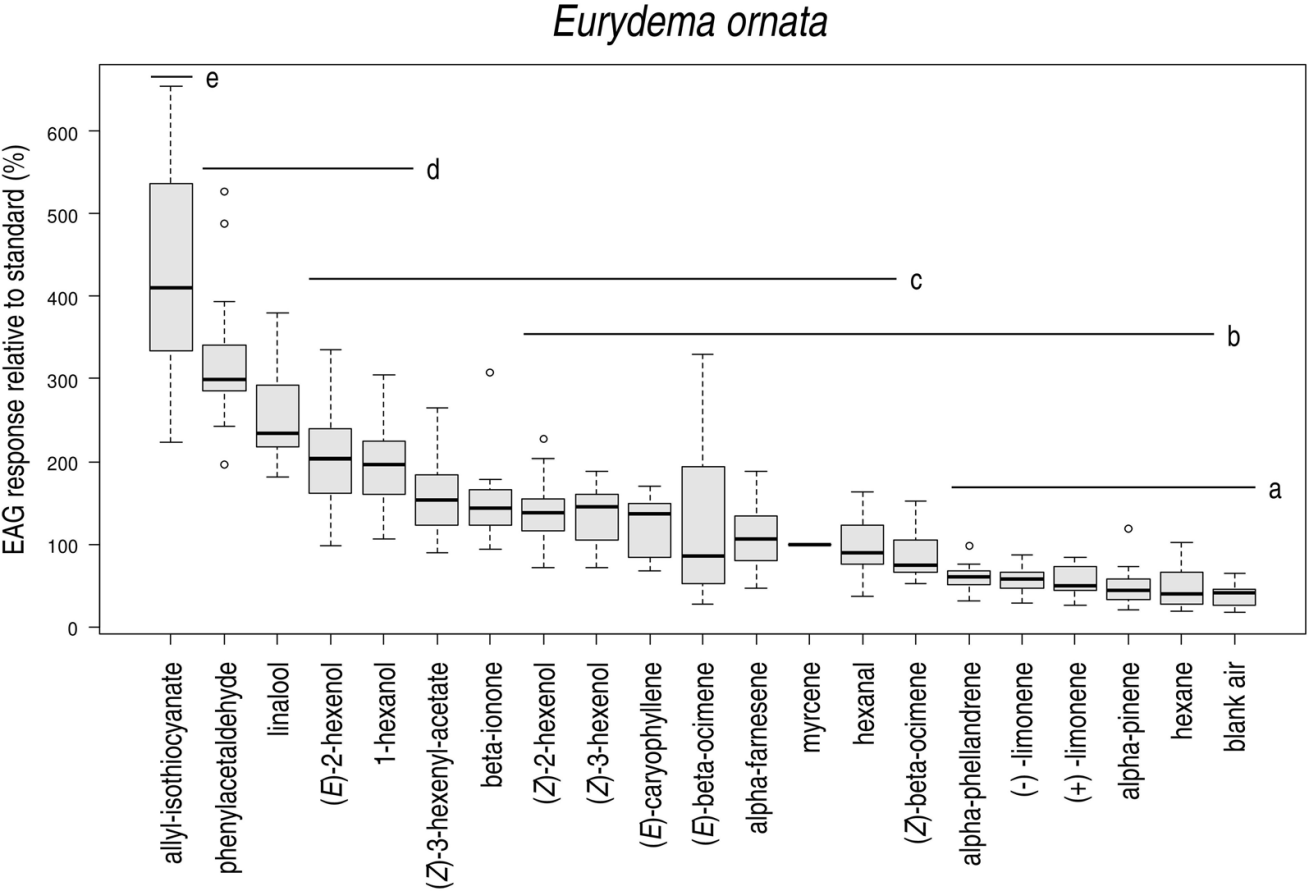
Electroantennographic responses of antennae of *Eurydema ornata* adults (N = 13) to plant volatiles relative to a standard (myrcene). Treatments marked with the same letter are not significantly different at p = 5% by Kruskal–Wallis test, pairwise comparison by Wilcoxon test with Benjamini–Hochberg correction.

Several plant volatiles elicited electroantennographic responses, different from solvent control (Fig. 1). Three compounds elicited significantly higher responses than the standard, these were the following: allyl isothiocyanate, phenylacetaldehyde and ± linalool. These compounds were tested in consecutive field experiments.

### Field experiments

In the course of the field experiments three *Eurydema* species were caught: *E. oleracea*, *E. ornata* and *E. ventralis*.

#### Field catches of *Eurydema ornata*

**Figure 2:**
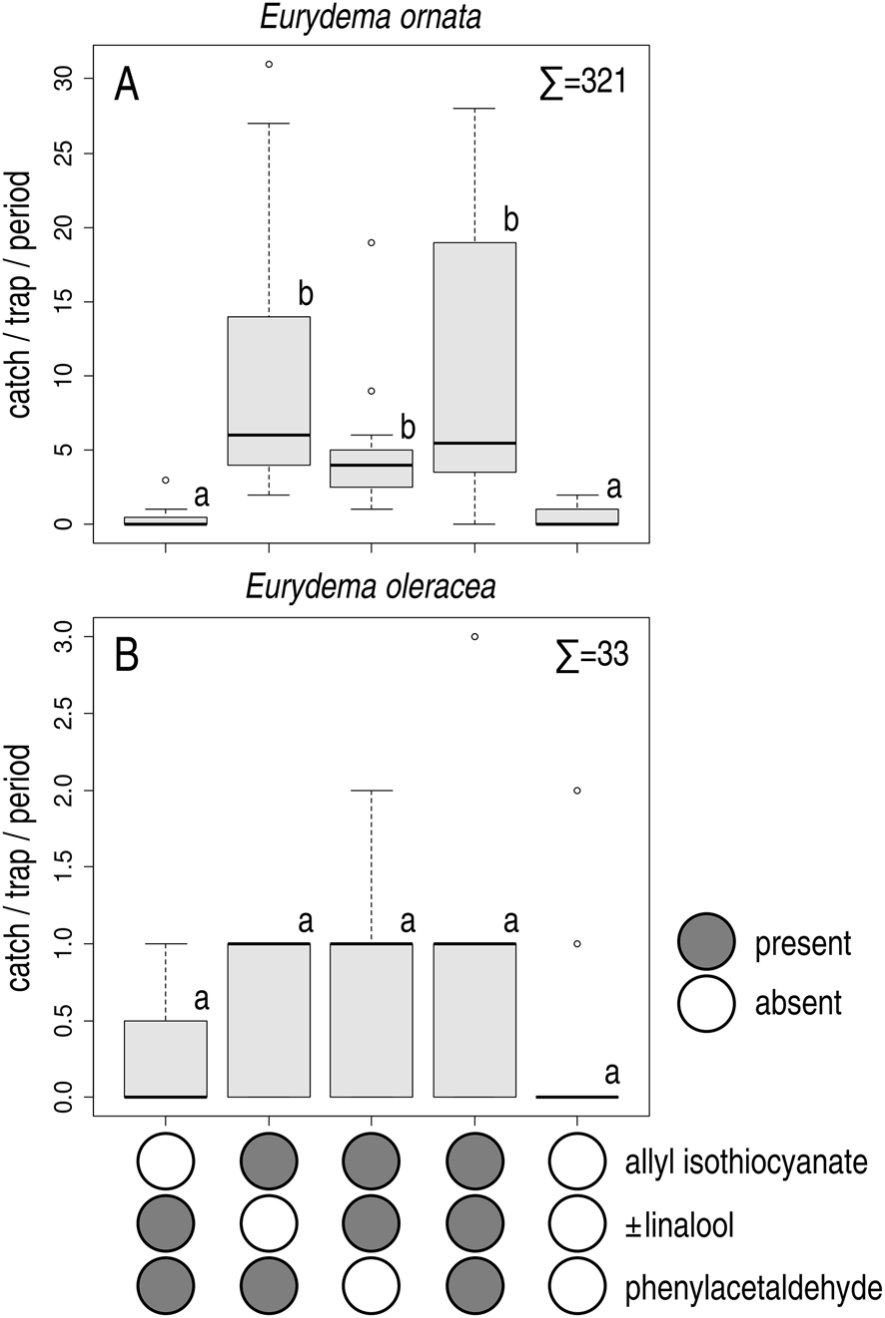
Field catches of *Eurydema ornata* (**A**) and *E. oleracea* (**B**) adults with binary and ternary combinations of ± linalool, phenylacetaldehyde and allyl isothiocyanate and in unbaited traps (Exp. 1). Treatments marked with the same letter are not significantly different within one diagram at p = 5% by Kruskal–Wallis test, pairwise comparison by Wilcoxon test with Benjamini–Hochberg correction.

In Experiment 1, only combinations containing allyl isothiocyanate caught more *E. ornata* than unbaited traps. The treatments containing allyl isothiocyanate did not differ significantly (Fig. 2A). Both males and females were caught (ratio of females 51.04%), with no apparent sex ratio differences between treatments.

**Figure 3:**
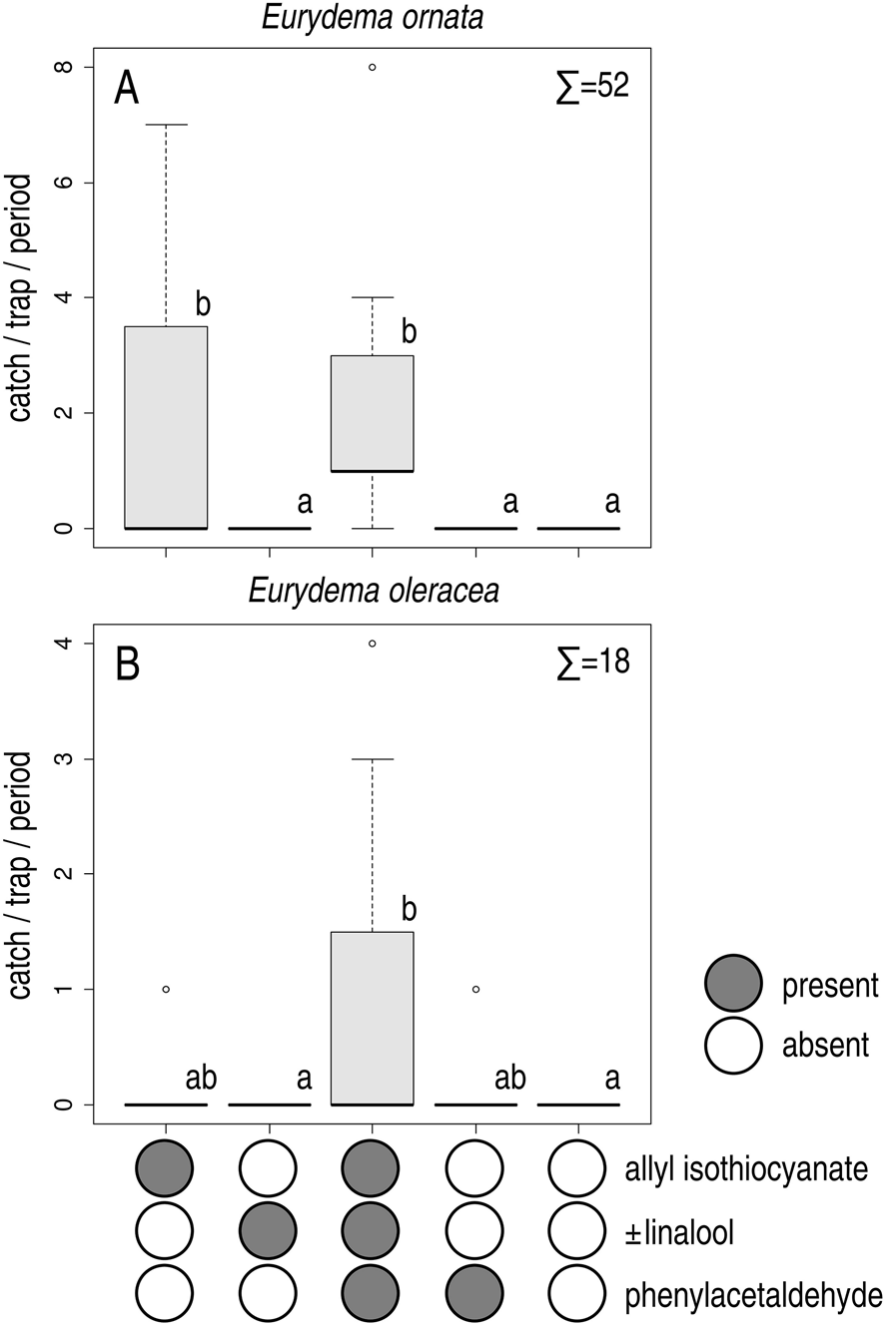
Field catches of *Eurydema ornata* (**A**) and *E. oleracea* (**B**) adults with ± linalool, phenylacetaldehyde, allyl isothiocyanate, their ternary combination and in unbaited traps (Exp. 2). Treatments marked with the same letter are not significantly different within one diagram at p = 5% by Kruskal–Wallis test, pairwise comparison by Wilcoxon test with Benjamini–Hochberg correction.

In Experiment 2, allyl isothiocyanate and the ternary combination of allyl isothiocyanate, phenylacetaldehyde and ± linalool attracted more *E. ornata* than unbaited traps. Unbaited traps and traps baited with either phenylacetaldehyde alone or ± linalool alone did not catch any individuals (Fig. 3A). Both males and females were caught (ratio of females 39.62%), with no apparent sex ratio differences between treatments.

**Figure 4:**
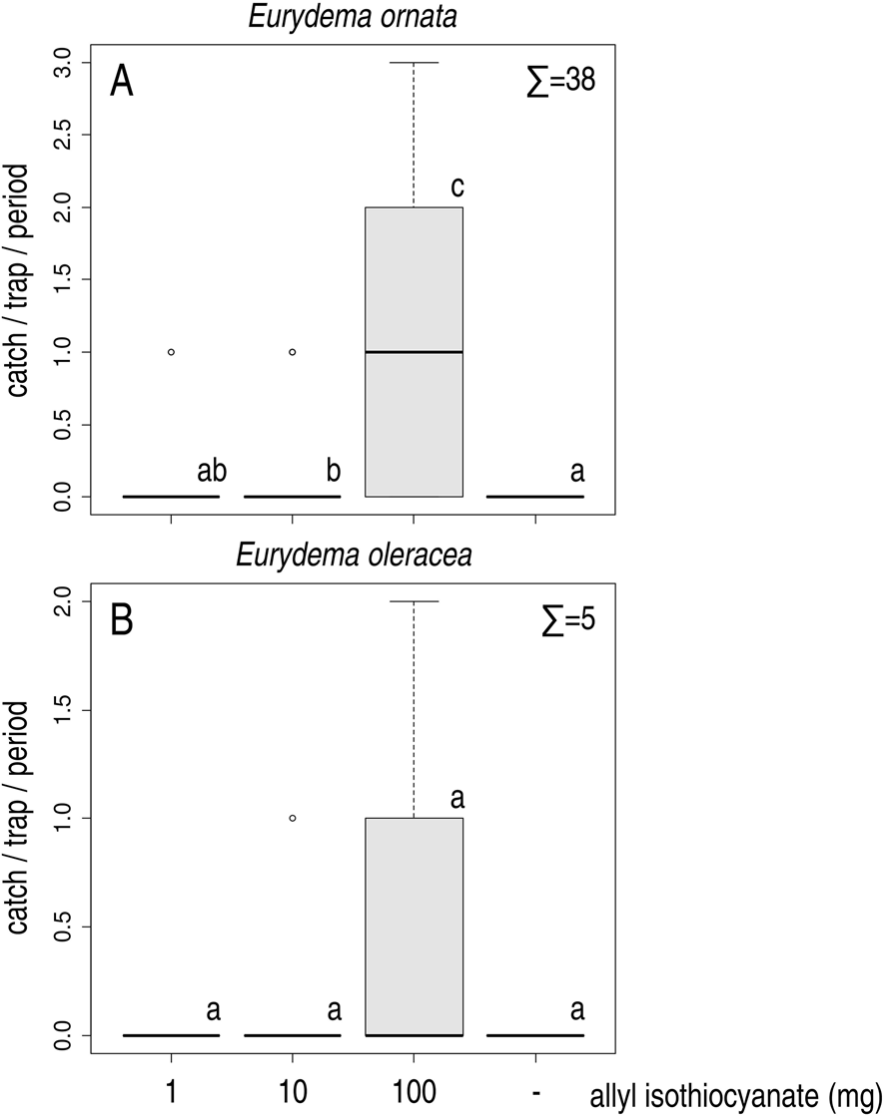
Field catches of *Eurydema ornata* (**A**) and *E. oleracea* (**B**) adults in traps baited with different doses of allyl isothiocyanate and in unbaited traps (Exp. 3). Treatments marked with the same letter are not significantly different at p = 5% by Kruskal–Wallis test, pairwise comparison by Wilcoxon test with Benjamini–Hochberg correction.

In Experiment 3, traps baited with 10 mg of allyl isothiocyanate caught more *E. ornata* than unbaited traps, however, catches did not differ from those of traps baited with 1 mg dose of the compound. On the other hand, traps baited with 100 mg of allyl isothiocyanate caught more than all other treatments. Unbaited traps did not catch any individuals (Fig. 4A). Again both males and females were caught (ratio of females 52.63%) with no apparent sex ratio differences between treatments. Catches showed a significant positive correlation with increasing dose, both for males (Spearman's rank correlation rho = 0.393, p < 0.001) and females (Spearman's rank correlation rho = 0.494, p < 0.001).

In Experiment 4, both allyl isothiocyanate and allyl isothiocyanate + phenylacetaldehyde caught more *E. ornata* than unbaited traps. Catches of the baited treatments did not differ significantly. Males and females responded similarly (Table 2).

**Table 2:** Mean catches of *Eurydema* spp. males and females with allyl isothiocyanate, phenylacetaldehyde + allyl isothiocyanate and in unbaited traps (Exp. 4).

Treatment	*E. ornata*		*E. oleracea*		*E. ventralis*	
	Males	Females	Males	Females	Males	Females
	Mean ± SE	Mean ± SE	Mean ± SE	Mean ± SE	Mean ± SE	Mean ± SE
Allyl isothiocyanate	0.64 ± 0.23 b	0.76 ± 0.23 b	0 ± 0 a	0.40 ± 0.24 a	0.10 ± 0.10 a	0 ± 0 a
Allyl isothiocyanate + phenylacetaldehyde	0.92 ± 0.32 b	1.20 ± 0.29 b	0.20 ± 0.20 a	0 ± 0 a	0.10 ± 0.10 a	0.20 ± 0.13 a
No bait	0.04 ± 0.04 a	0 ± 0 a	0 ± 0 a	0 ± 0 a	0.10 ± 0.10 a	0 ± 0 a

Columns marked with the same letter are not significantly different at p = 5% by Kruskal–Wallis test, pairwise comparison by Wilcoxon test with Benjamini–Hochberg correction.

#### Field catches of *Eurydema oleracea*

In Experiment 1, catches of *E. oleracea* in different treatments did not differ significantly (Fig. 2B). Both males and females were caught (ratio of females 55.88%), with no apparent sex ratio differences between treatments.

In Experiment 2, traps baited with the ternary combination attracted more *E. oleracea* than unbaited traps and than traps baited with ± linalool, neither of which caught any individuals of this species. On the other hand, catches with the ternary combination did not differ from those baited with either allyl isothiocyanate or phenylacetaldehyde (Fig. 3B). Both males and females were caught (ratio of females 72.22%), with no apparent sex ratio differences between treatments.

In Experiment 3, only a few *E. oleracea* individuals were caught. Only traps baited with either 10 or 100 mg of allyl isothiocyanate caught *E. oleracea*, however, treatments did not differ significantly (Fig. 4B). Both males and females were caught (ratio of females 80%), with no apparent sex ratio differences between treatments. Due to the low catches correlations were not calculated.

In Experiment 4, again only few *E. oleracea* were caught, treatments did not differ significantly. Both males and females were caught (Table 2).

#### Field catches of *Eurydema ventralis*

In the experiments *E. ventralis* was only caught in Experiment 4, nevertheless, the species was represented in low numbers, treatments did not differ significantly. Both males and females were caught (Table 2).

## Discussion

In the current study *E. ornata* was found to be attracted to baits containing allyl isothiocyanate. To our knowledge this is the first report on field attraction of an *Eurydema* species to a synthetic semiochemical. As for other pentatomids, Thrift et al.^22^ found higher abundance of *Murgantia histrionica* (Hahn) on plants baited with either allyl isothiocyanate or benzyl isothiocyanate as compared to control plants with benzyl isothiocyanate showing more pronounced effect, and although the authors reported weak attraction to these plant volatiles, the compounds further increased the attractive effect of the pheromone.

Reports on field attraction of pentatomids to plant volatiles are scarce^3^. For instance, for *Halyomorpha halys* (Stal) plant volatiles were found to be only slightly attractive or even inhibitory^23^. Furthermore, it is rather commonly reported in pentatomids that although the pheromone blends show activity, several species were found to rarely enter traps^4^. This is possibly due to the importance of vibratory signals in their intraspecific communication^24^. For instance, Thrift et al.^22^ applied baited plants in their studies. In the current study bugs were attracted to the baits and were caught in the traps, which is especially promising in respect of development of tools for potential practical applications in the future. Optimisation of bait composition and trap design may result in further enhanced effectiveness of the traps.

According to the literature *E. ornata* primarily feeds on generative parts of plants^11,16^. However, in our research, common floral volatiles, phenylacetaldehyde and ± linalool, present also in cruciferous plants^25^, despite their electroantennographic activity, did not elicit behavioral response in field experiments, when presented on their own. Furthermore, these compounds did not influence activity of allyl isothiocyanate considerably.

Breakdown products of glucosinolates are characteristic for cruciferous plants^26^. Thiocyanates and isothiocyanates were found to attract pests of brassicaceous crops, for instance, allyl isothiocyanate is a known attractant for *Phyllotreta* spp. (e.g.^19,27^).

In the current research three *Eurydema* species were caught, however, *E. oleracea* and *E. ventralis* were usually represented by few individuals in trap catches, mostly in insufficient numbers for statistical evaluation. However, research targeting these pests may bring fruitful results, as it was found in previous studies that even closely related species with overlapping host plant ranges may respond differently to plant volatiles (e.g.^19,28^). Therefore, other compounds characteristic for cruciferous plants may also be of interest, for instance for *Phyllotreta* spp. it was found that for some species, other isothyocyanates were more attractive than allyl isothyocyanate (e.g.^27^).

Furthermore, research on combinations of semiochemicals may also bring novel results as combinations of multiple stimuli may elicit more pronounced behavioural response than individual compounds^29^.

## Conclusions

Pentatomidae is a highly species rich group of true bugs, with considerable diversity in their chemical ecology^3,4^. As several species are important pests of various crops, tools for monitoring or possibly for direct control are of high potential value for agricultural practice.^4^

As research on chemical ecology of Pentatomidae primarily focuses on identification of pheromones, studies on attraction of pentatomids to plant volatiles are scarce^3^. To our knowledge, this is the first report of attraction of an *Eurydema* species to a semiochemical and one of the few examples of attraction of a pentatomid bug to a synthetic plant volatile in the field. The results may provide a first step in the development of practically applicable lures for trapping *E. ornata,* which may open perspectives for developments aiming applications in agricultural practice in the future. Furthermore, the results suggest that plant volatiles may provide prospects for practical applications for other pentatomid pests as well.

## Data Availability

The datasets used and/or analysed during the current study available from the corresponding author on reasonable request.
